# Supplementary material for: Evolutionary histories and antimicrobial resistance in Shigella flexneri and Shigella sonnei in Southeast Asia
Source: Commun Biol. 2021 Mar 19;4:353. doi: 10.1038/s42003-021-01905-9 (PMC7979695; doi:10.1038/s42003-021-01905-9)
Supplement: Supplementary file 2 — Supplementary Information [file 42003_2021_1905_MOESM2_ESM.pdf]

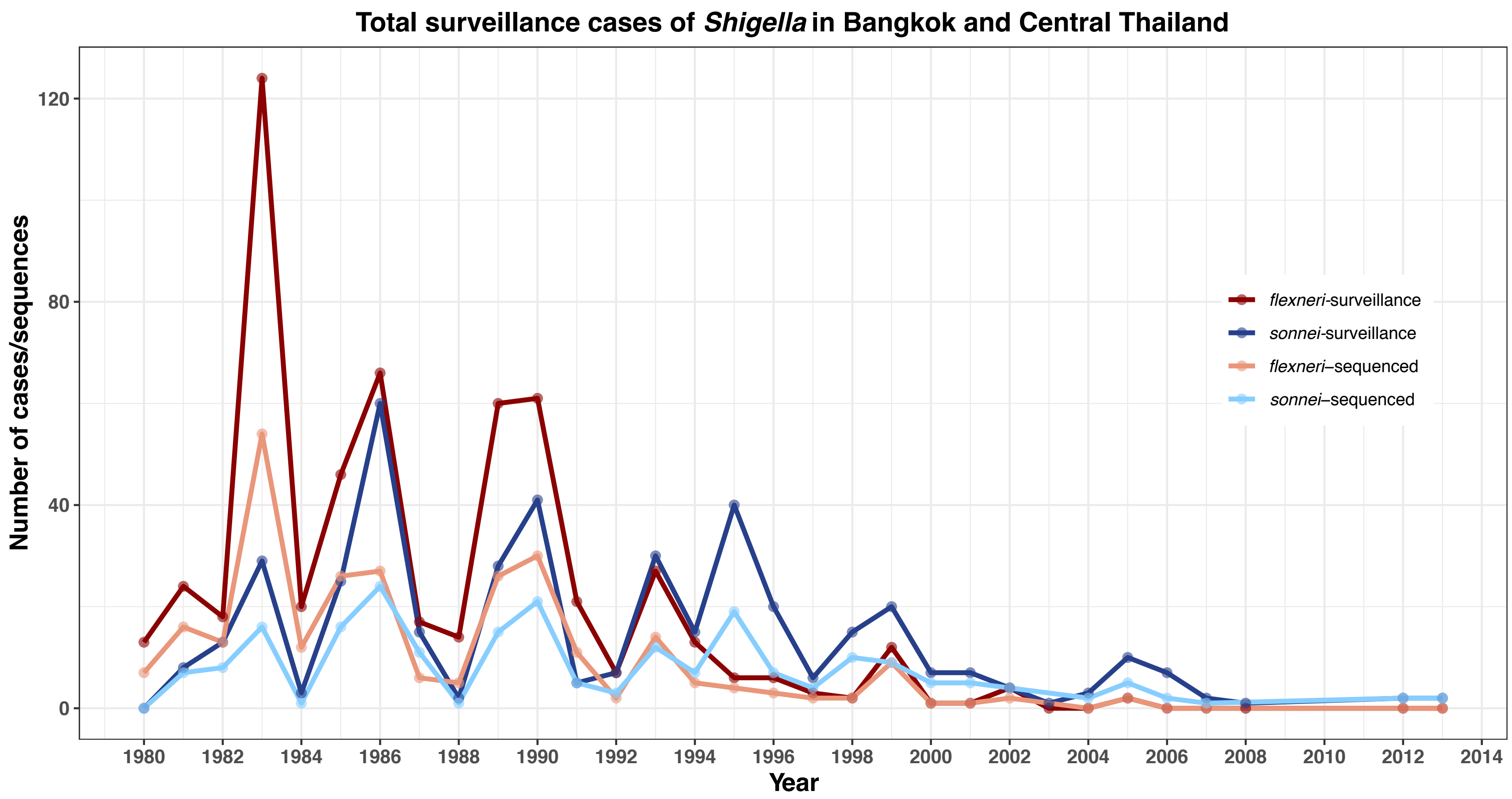

Supplementary Figure 1 The temporal trends of *Shigella* epidemiology and sequencing data in Bangkok and Central Thailand. *Shigella* epidemiology data are as recorded by AFRIMS, Bangkok, Thailand.

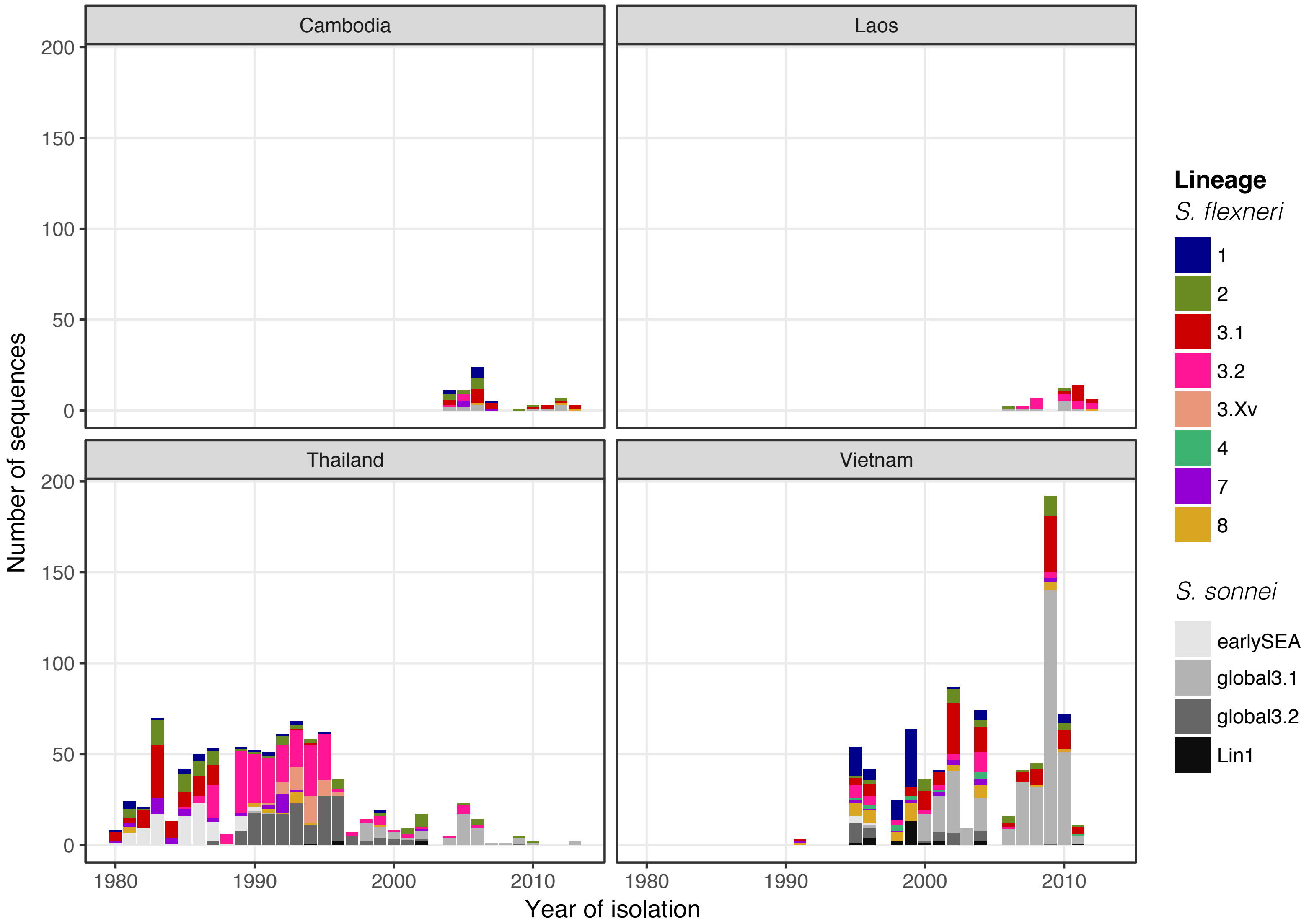

Supplementary Figure 2 Summary of *Shigella* sampling timeframe employed in this study. The number of sequences belonging to each *Shigella* lineage is plotted per calendar year for each Southeast Asian country (see Legend). Samples were sourced from multiple diarrhoea surveillance studies.

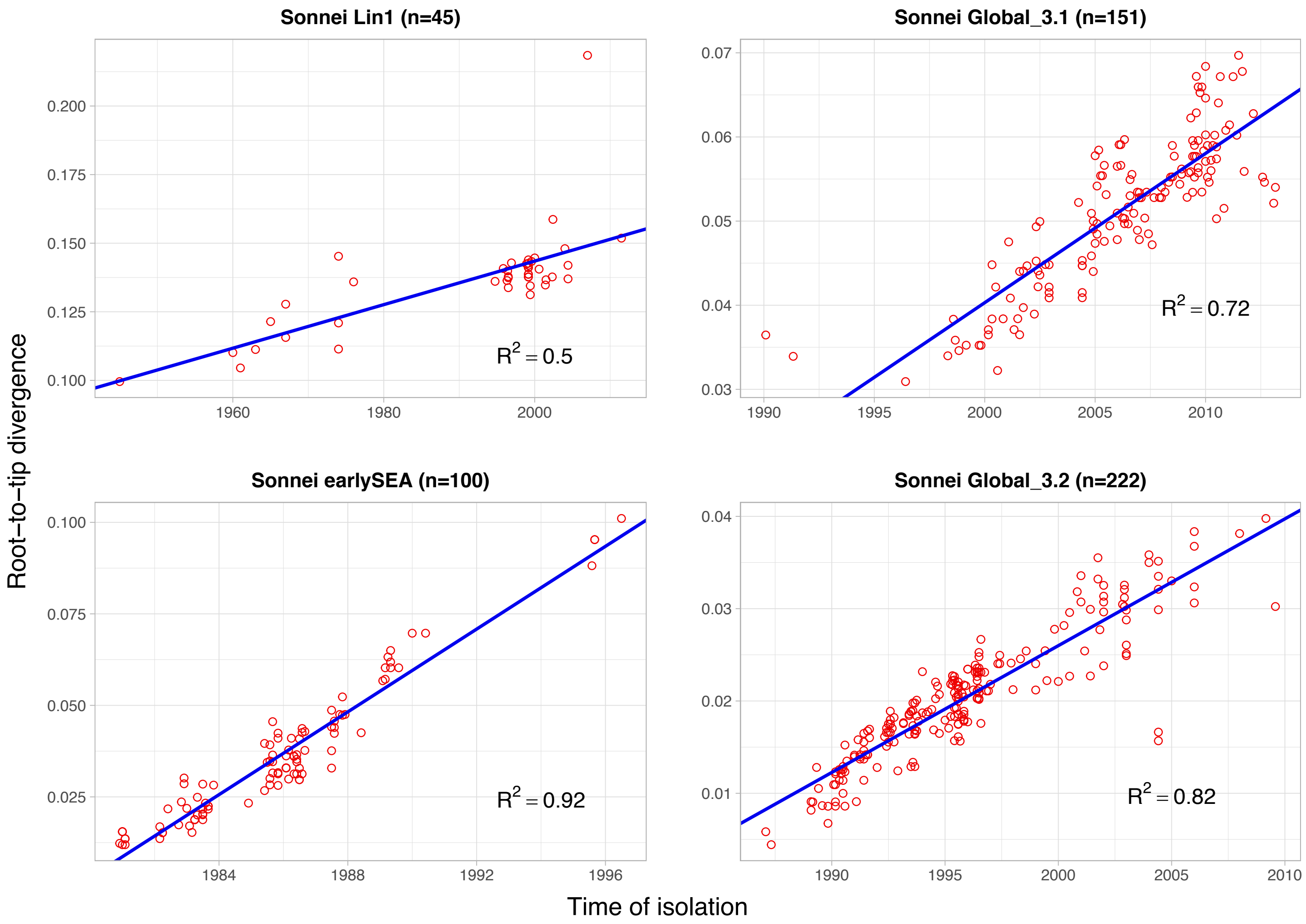

Supplementary Figure 3 Temporal phylogenetic signal for *Shigella sonnei*. The figure shows the correlations between the time of isolation (in months and years; x-axis) and root-to-tip divergence (y-axis) for *S. sonnei* Lineage 1 and three sublineages of Lineage 3. The linear regression line is coloured blue, and the coefficient of determination (R^2) is shown for each plot. Estimation of temporal signal was performed by TempEst v1.5.1, using the reference mapped SNP-based maximum likelihood phylogeny of each *S. sonnei* lineage and sublineage.

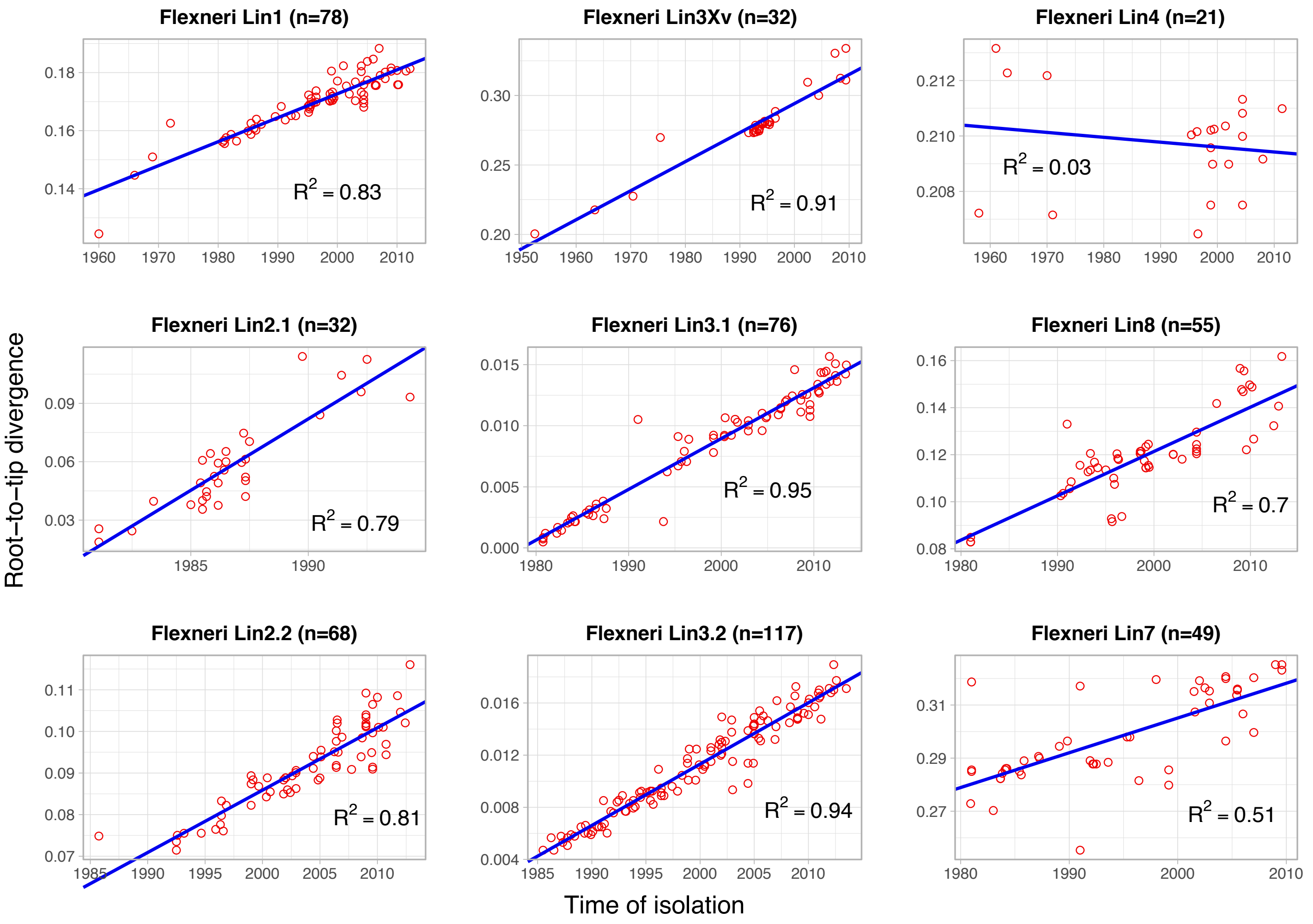

Supplementary Figure 4 Temporal phylogenetic signal for *Shigella flexneri*. The figure shows the correlations between the time of isolation (in months and years; x-axis) and root-to-tip divergence (y-axis) for each *S. flexneri* lineage or sublineage. The linear regression line is coloured blue, and the coefficient of determination (R^2) is shown for each plot. Estimation of temporal signal was performed using TempEst v1.5.1, using the reference mapped SNP-based maximum likelihood phylogeny of each *S. flexneri* lineage or sublineage.

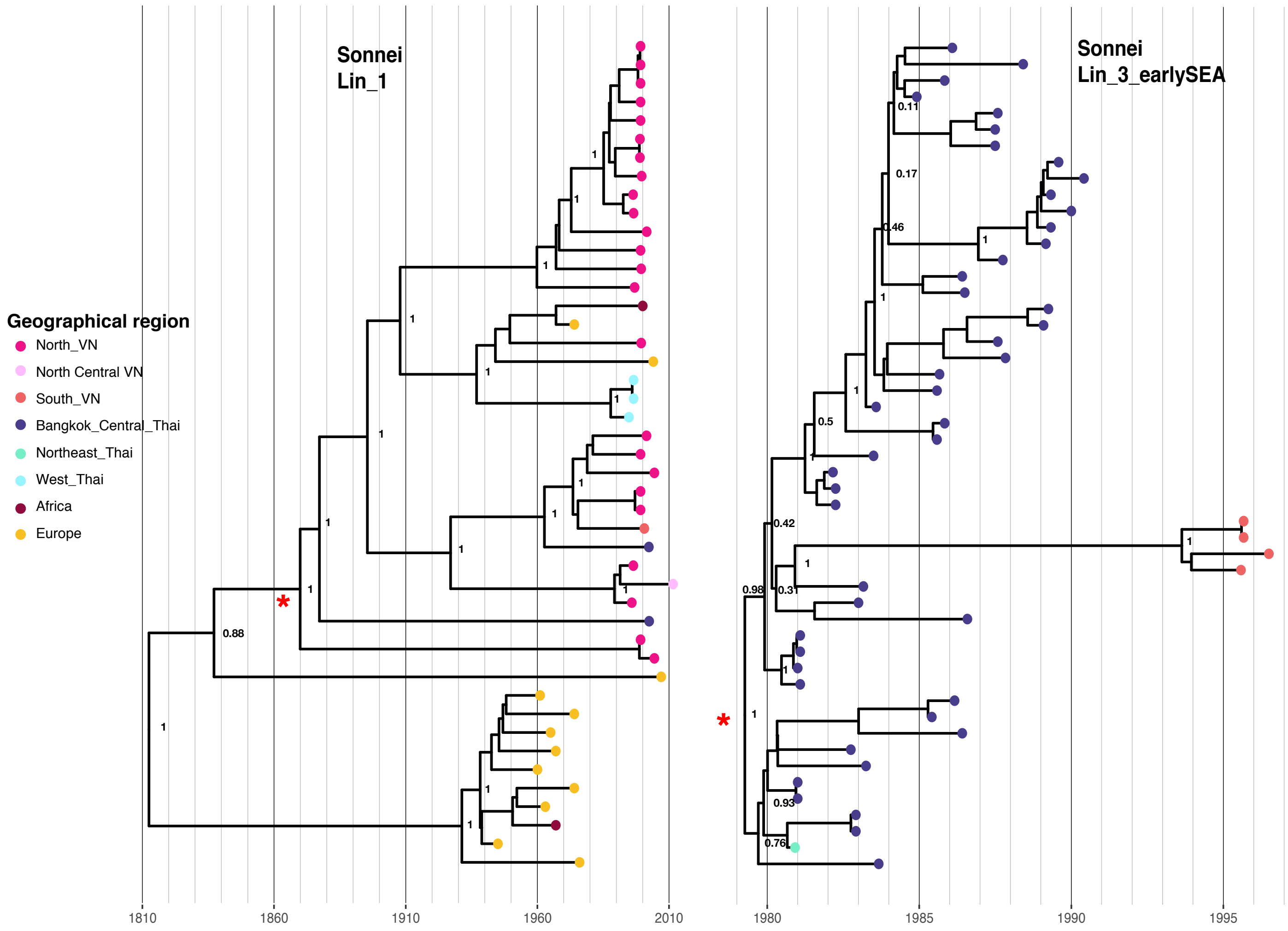

Supplementary Figure 5 Temporal phylogenetic reconstructions of *S. sonnei* Lineage 1 and Lineage 3 clade earlySEA. The figure shows the maximum clade credibility (MCC) phylogenies of 45 *S. sonnei* Lineage 1 and 51 sublineage earlySEA. The tip colours indicate the geographical origins of the isolates (see Legend). Posterior probability support is indicated on major internal nodes. The red asterisk delineates the time to most recent common ancestor (TMRCA) of Southeast Asian *Shigella* isolates.

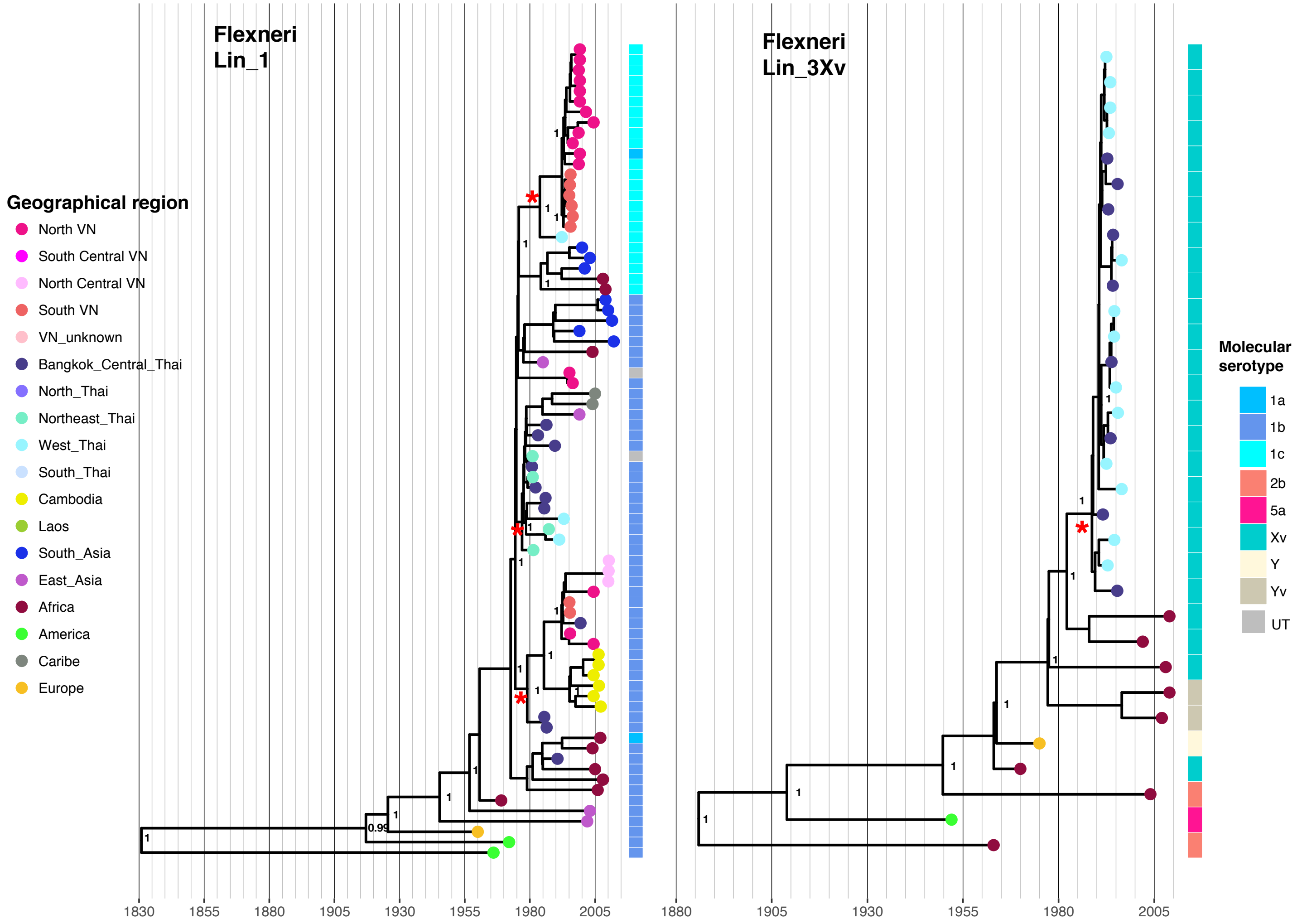

Supplementary Figure 6 Temporal phylogenetic reconstructions of *S. flexneri* Lineage 1 and 3Xv. The figure shows MCC phylogenetic reconstructions of 78 *S. flexneri* Lineage 1 and 32 sublineage 3Xv isolated in Southeast Asia. The leaf colours specify the geographical origins while the column to the right of the tree indicates the molecular serotype of each isolate (see Legend). UT: Untypeable. Posterior probability support is indicated on major internal nodes. The red asterisk delineates major Southeast Asian *S. flexneri* populations (Lin-1) or TMRCA of Southeast Asian isolates (Lin-3Xv)

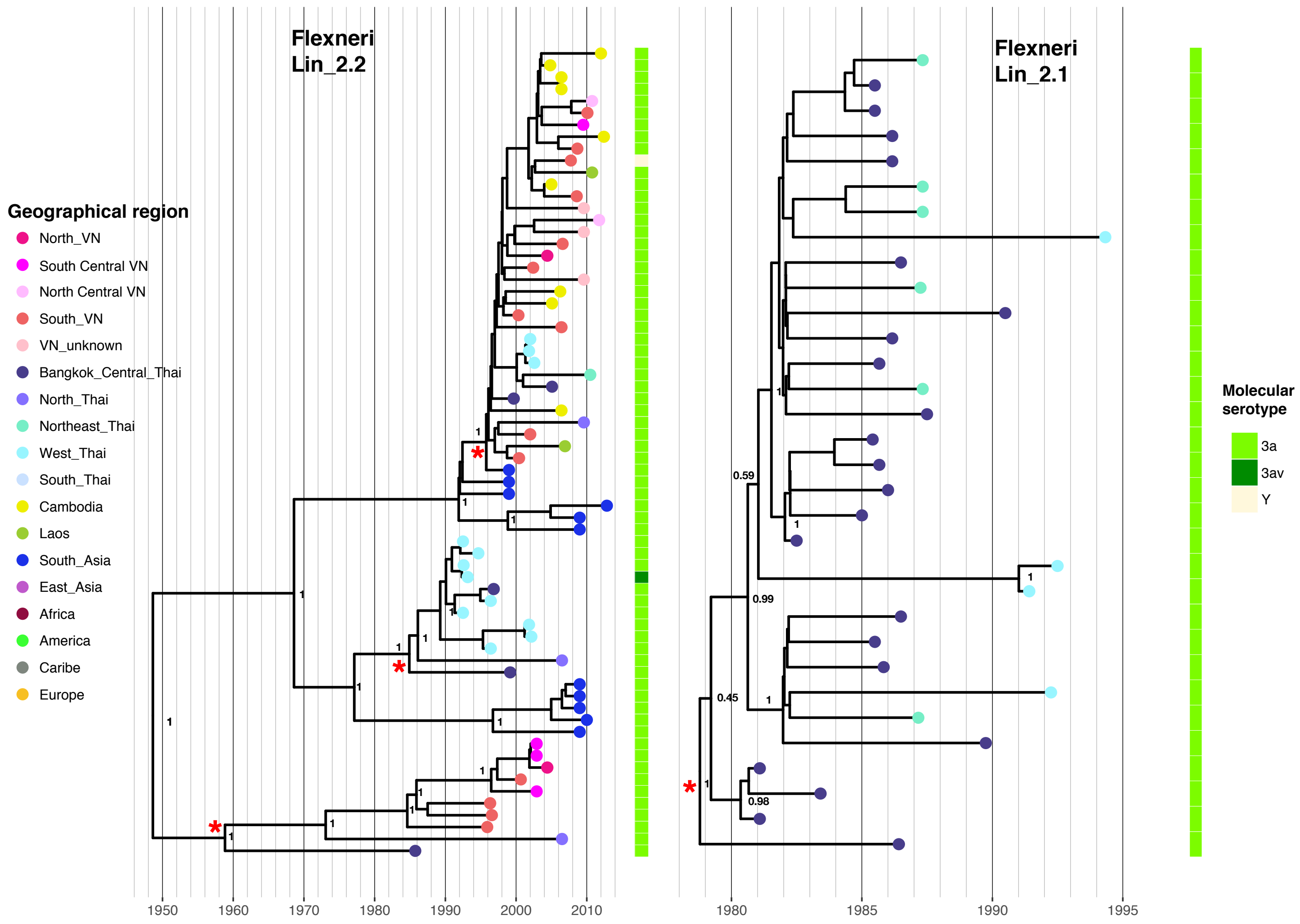

Supplementary Figure 7 Temporal phylogenetic reconstructions of *S. flexneri* Lineage 2. The figure shows the MCC phylogenies of 32 *S. flexneri* Lineage 2.1 and 68 Lineage 2.2 isolated in Southeast Asia. The leaf colours specify the geographical origins while the column to the right of the tree indicates the molecular serotype of each isolate, as assessed by genomic analyses (see Legend). Posterior probability support is indicated on major internal nodes. The red asterisk delineates major Southeast Asian *S. flexneri* populations (Lin-2.2) or TMRCA of Southeast Asian isolates (Lin-2.1).

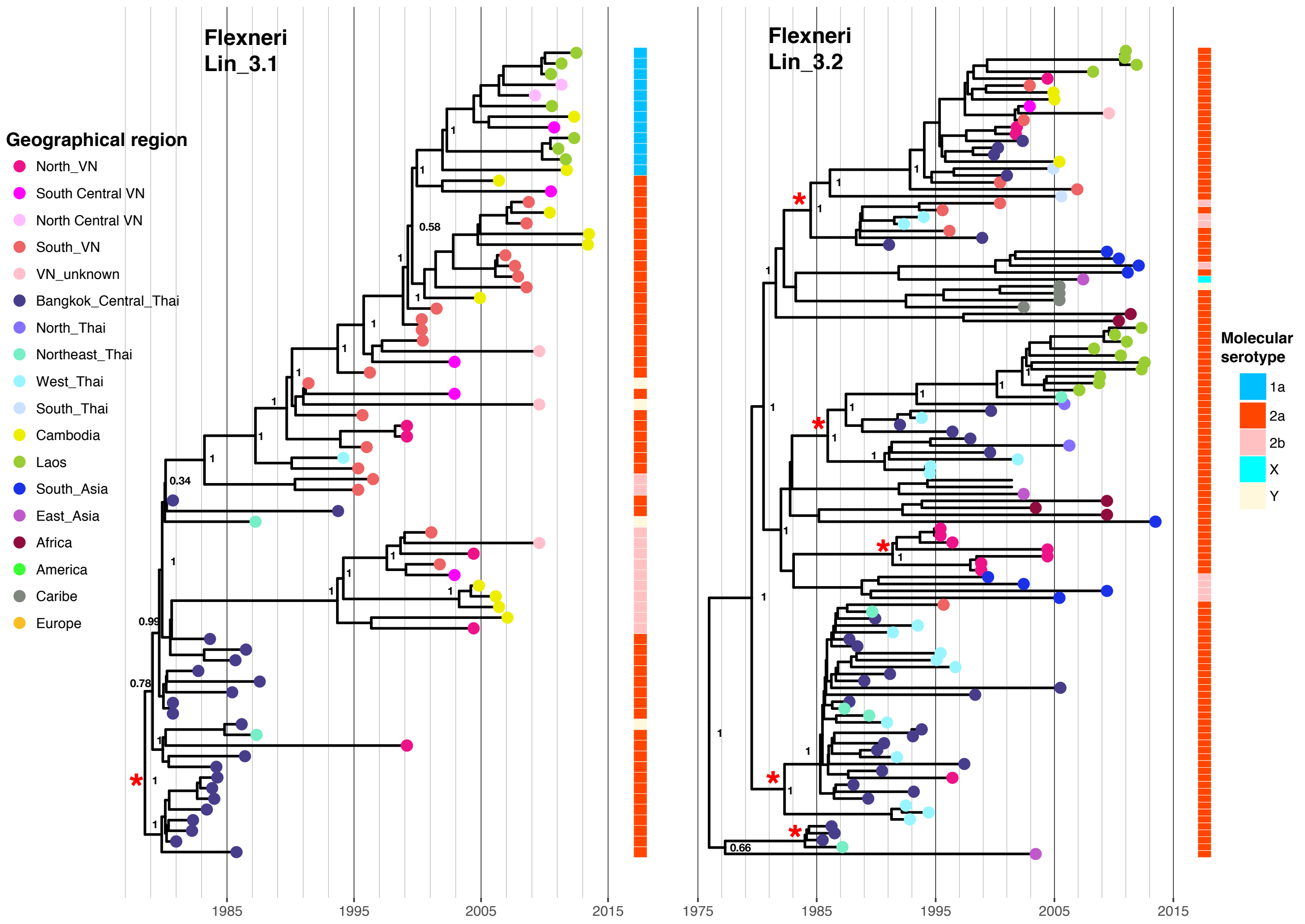

Supplementary Figure 8 Temporal phylogenetic reconstructions of *S. flexneri* Lineage 3. The figure shows the MCC phylogenies of 76 *S. flexneri* Lineage 3.1 and 117 Lineage 3.2 isolated in Southeast Asia. The leaf colours specify the geographical origins while the column to the right of the tree indicates the molecular serotype of each isolate, as assessed by genomic analyses (see Legend). Posterior probability support is indicated on major internal nodes. The red asterisk delineates major Southeast Asian *S. flexneri* populations (Lin-3.2) or TMRCA of Southeast Asian isolates (Lin-3.1). Absence of tip label indicates missing geographical information.

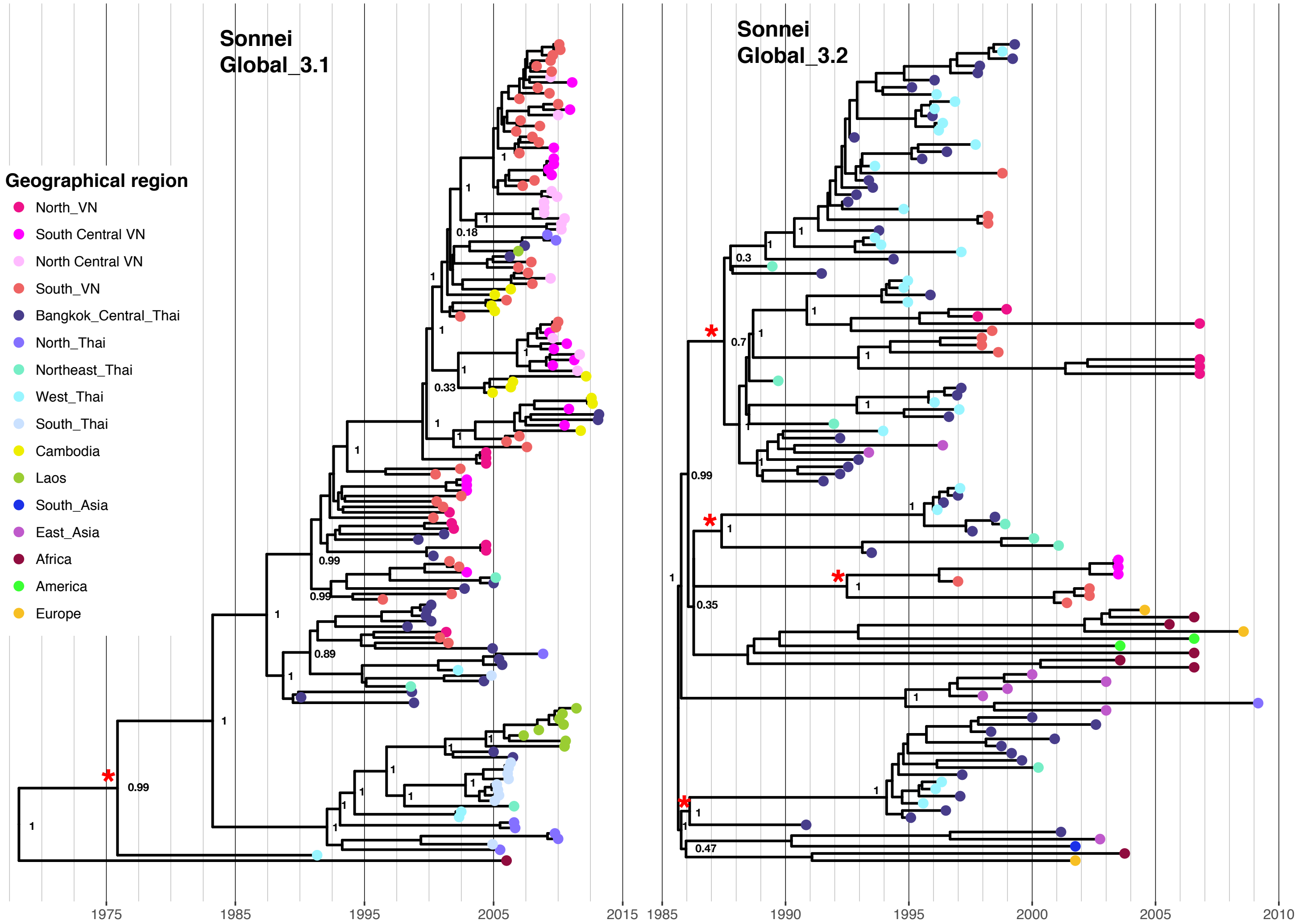

Supplementary Figure 9 Temporal phylogenetic reconstructions of *S. sonnei* Lineage 3. The figure shows the MCC phylogenies of 151 *S. sonnei* sublineages Global 3.1 and 117 Global 3.2. The tip colours indicate the geographical origins of the isolates (see Legend). Posterior probability support is indicated on major internal nodes. The red asterisk delineates major Southeast Asian *S. sonnei* populations (Global 3.2) or TMRCA of Southeast Asian isolates (Global 3.1).

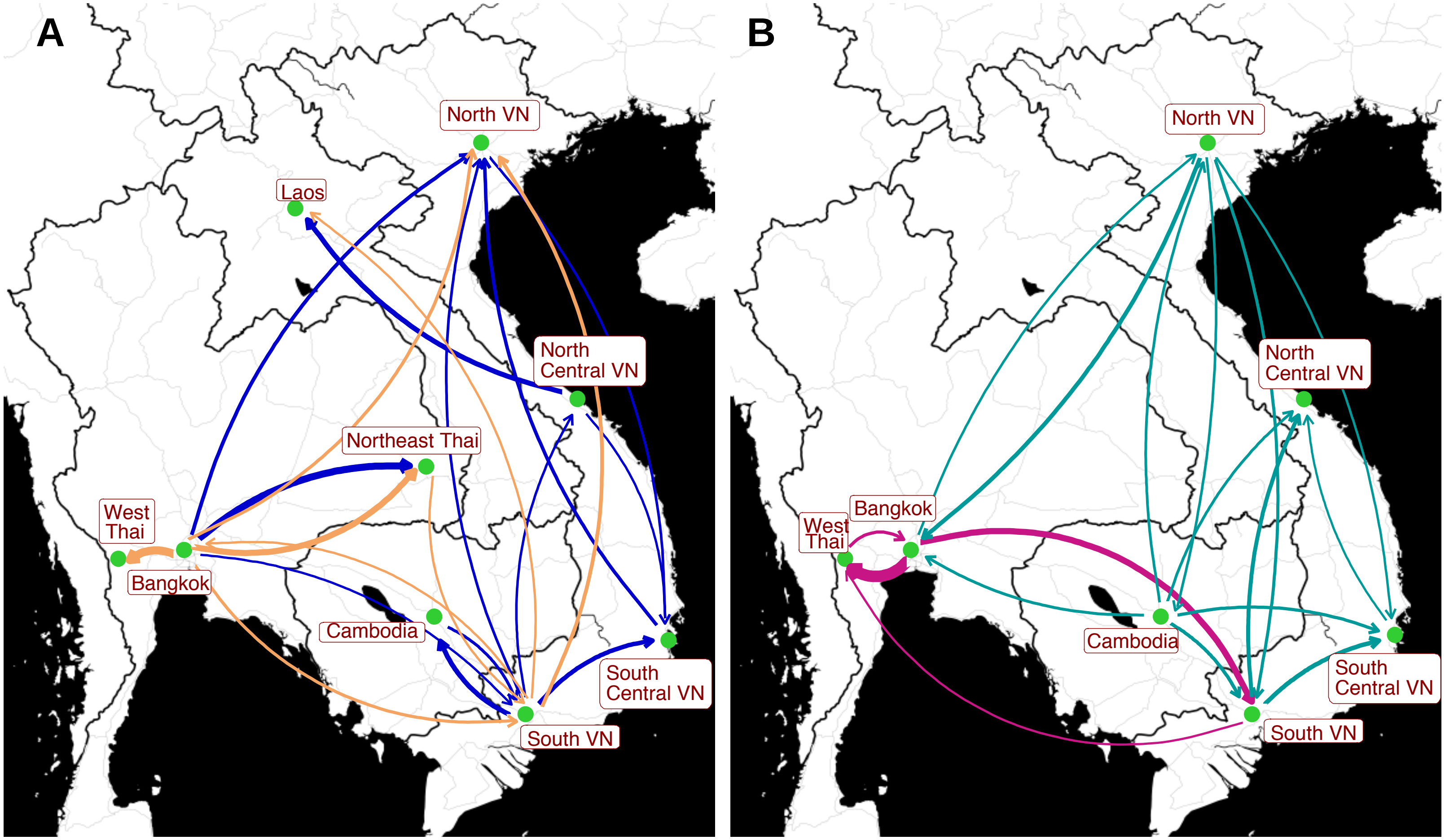

Supplementary Figure 10 Migration network of *Shigella* within Southeast Asia. The figures summarize the geographical transitions of (A) the two *S. flexneri* Lineages, 3.1 (blue arrows) and 3.2 (orange arrows); and (B) the two *S. sonnei* sublineages, Global 3.1 (dark cyan arrows) and Global 3.2 (violet arrows). Each region is highlighted by a green circle on the map, with the capitals Vientiane and Phnom Penh selected as representatives for Laos and Cambodia due to limited samples. The width of the arrows is proportional to the number of transition events between geographical states, which were inferred by stochastic mapping of geographical traits (see Methods). Transition events less than one are considered non-significant and are not represented in this figure. The Southeast Asia map

was created using the R package ‘ggmap’ with the following coordinates (longitude=103.29291, latitude=15.203422).

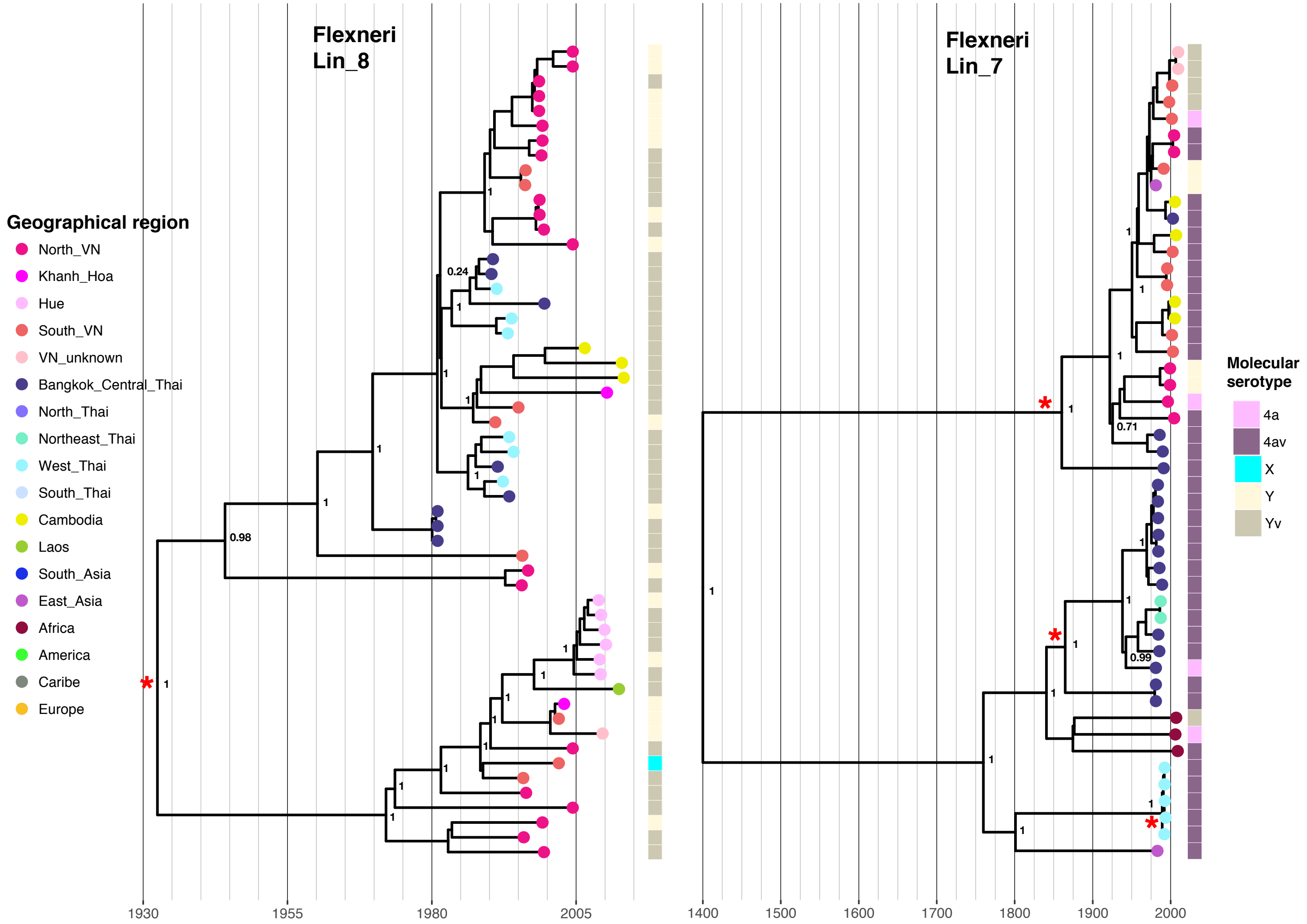

Supplementary Figure 11 Temporal phylogenetic reconstructions of *S. flexneri* Lineages 7 and 8. The figure shows the MCC phylogenies of 49 *S. flexneri* Lineage 7 and 55 Lineage 8 isolated in Southeast Asia. The leaf colours specify the geographical origins while the column to the right of the tree indicates the

molecular serotype of each isolate, as assessed by genomic analyses (see Legend). Posterior probability support is indicated on major internal nodes. The red asterisk delineates major Southeast Asian *S. flexneri* populations (Lin-7) or TMRCA of Southeast Asian isolates (Lin-8).

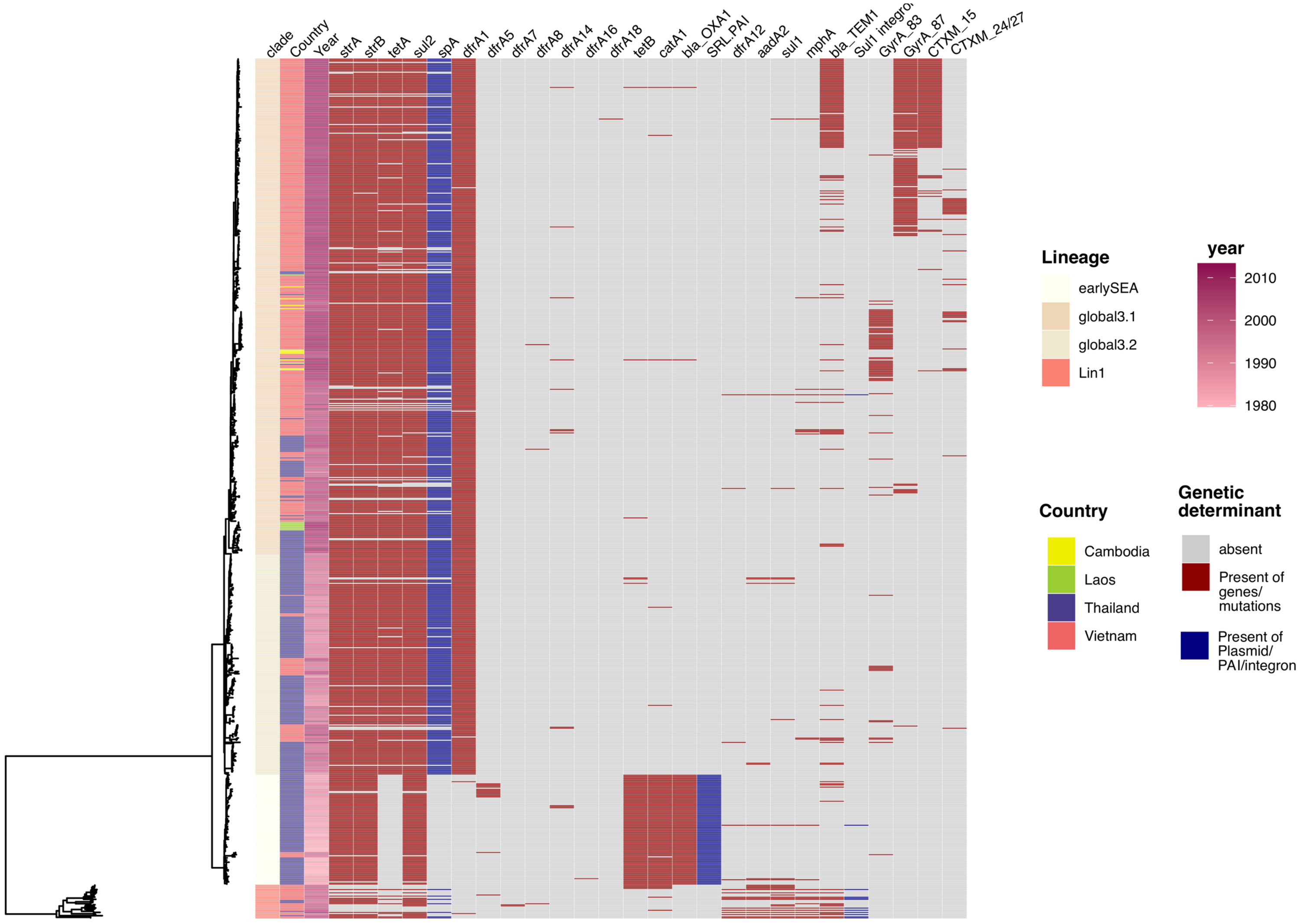

Supplementary Figure 12 The resistome of 789 *Shigella sonnei* isolated in Southeast Asia. The phylogeny on the left is subset from the maximum likelihood phylogeny of *S. sonnei* presented in Figure 2B. The metadata for each isolate are shown on the right, including its phylogenetic lineage, country and year of

isolation, together with major antimicrobial resistance determinants (gene or mutation) and relevant genomic material harbouring these determinants (SRL-PAI, plasmid spA, and *sul1* MDR integron) (See key). Missing data are denoted in white cells.

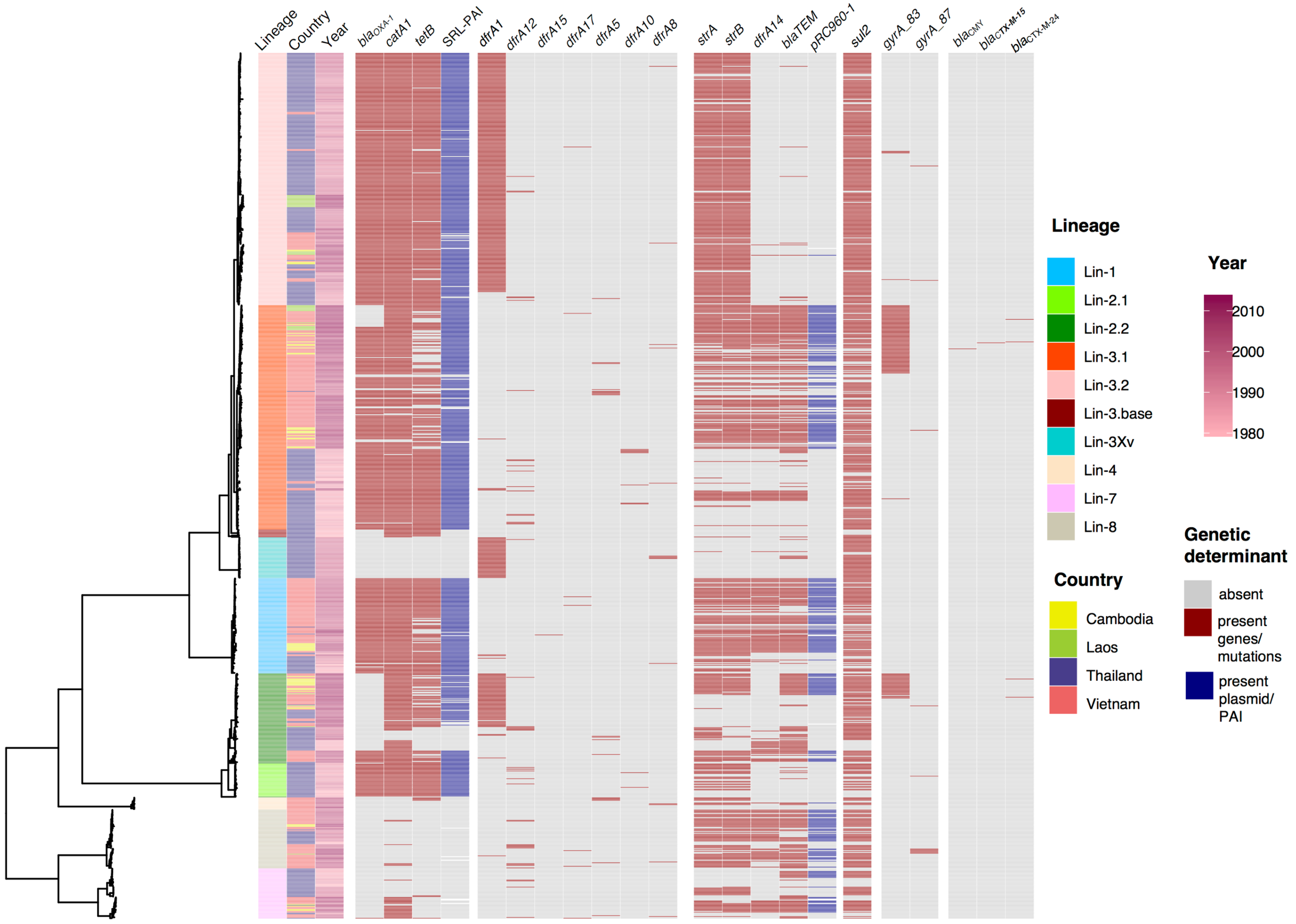

Supplementary Figure 13 The resistome of 999 *Shigella flexneri* isolated in Southeast Asia. The phylogeny on the left is subset from the maximum likelihood phylogeny of *S. flexneri* presented in Figure 2A. The metadata for each isolate are shown on the right, including its phylogenetic lineage, country and year of

isolation, together with major antimicrobial resistance determinants (gene or mutation) and relevant genomic material harbouring these determinants (SRL-PAI and plasmid pRC960-1) (See key). Missing data are denoted in white cells.

**Region**	**Timespan**	***S. flexneri***	***S. sonnei***	**Inclusive areas**
Bangkok and Central Thailand	1980-2013	280	227	Bangkok, Nonthaburi, Samut Sakhon, Nakhon Pathom
Northern Thailand	2005-2010	7	9	Chiang Rai, Mae Hong Son
Northeast Thailand	1980-2010	55	12	Nong Khai, Ubon Ratchathani, Surin, Nakhon Ratchasima
Western Thailand	1990-2002	186	82	Tak, Kanchanaburi, Ratchaburi
Southern Thailand	2004-2006	3	16	Trang
Cambodia	2004-2013	56	13	Phnom Penh, Siem Reap
Laos	2006-2012	34	9	Vientiane
Northern Vietnam	1995-2004	147	56	Hanoi
North Central Vietnam	2008-2011	28	49	Hue
South Central Vietnam	2002-2011	41	86	Khanh Hoa
Southern Vietnam	1991-2010	162	246	Ho Chi Minh City, Dong Thap, Tien Giang, and Ben Tre

Supplementary Table 1 Summary of isolates used in Southeast Asia *Shigella* evolution analysis

gtr (glucosylation)	***oac*** (O-acetylation)	***opt*** (phosphorylation)	**Genomic serotype**
gtrI			1a
gtrI	*oacA*		1b
gtrI + gtrIc			1c
gtrII	*oacD*		2a
gtrII + gtrX			2b
gtrII		*opt*	2av
gtrX	*oacA*		3a
	oacA		3b
gtrIV			4a
gtrIV	*oacA*		4b
gtrIV		*opt*	4av
gtrV			5a
gtrV + gtrX			5b
gtrX			X
gtrX		*opt*	Xv
			Y
		opt	Yv

Supplementary Table 2 Geno-serotyping scheme for *Shigella flexneri*
