# Supplementary material for: Evolutionary histories and antimicrobial resistance in Shigella flexneri and Shigella sonnei in Southeast Asia
Source: Commun Biol. 2021 Mar 19;4:353. doi: 10.1038/s42003-021-01905-9 (PMC7979695; doi:10.1038/s42003-021-01905-9)
Supplement: Supplementary file 3 — Description of Additional Supplementary Files [file 42003_2021_1905_MOESM3_ESM.pdf]

Description of Additional Supplementary Files

File Name: Supplementary Data 1

Description: Source data for Figure 1, Figure 3a, Figure 3b, Figure 4a, Figure 4b, Figure 4c, and Figure 5 are included in individual sheets as labeled.
